# Learning to learn: Single session acquisition of new rules by freely moving mice

**DOI:** 10.1093/pnasnexus/pgae203

**Published:** 2024-05-19

**Authors:** Amir Levi, Noam Aviv, Eran Stark

**Affiliations:** Department of Physiology and Pharmacology, Faculty of Medicine, Tel Aviv University, Tel Aviv 6997801, Israel; Sagol School of Neuroscience, Tel Aviv University, Tel Aviv 6997801, Israel; Department of Physiology and Pharmacology, Faculty of Medicine, Tel Aviv University, Tel Aviv 6997801, Israel; Department of Physiology and Pharmacology, Faculty of Medicine, Tel Aviv University, Tel Aviv 6997801, Israel; Sagol School of Neuroscience, Tel Aviv University, Tel Aviv 6997801, Israel; Sagol Department of Neurobiology, Haifa University, Haifa 3103301, Israel

**Keywords:** animal cognition, associative learning, hybrid mice, reference memory, visual discrimination

## Abstract

Learning from examples and adapting to new circumstances are fundamental attributes of human cognition. However, it is unclear what conditions allow for fast and successful learning, especially in nonhuman subjects. To determine how rapidly freely moving mice can learn a new discrimination criterion (DC), we design a two-alternative forced-choice visual discrimination paradigm in which the DCs governing the task can change between sessions. We find that experienced animals can learn a new DC after being exposed to only five training and three testing trials. The propensity for single session learning improves over time and is accurately predicted based on animal experience and criterion difficulty. After establishing the procedural learning of a paradigm, mice continuously improve their performance in new circumstances. Thus, mice learn to learn.

**Significance Statement**

Humans excel at learning from examples and adapting to new circumstances, but conditions for efficient learning in nonhuman subjects are unclear. In this study, we explore the adaptability of mice to new circumstances using a visual discrimination task. We find that mice can learn a new discrimination criterion (DC) within a single session, a capacity that enhances with experience and varies with DC difficulty. Furthermore, mice exhibit flexibility in learning strategy based on the physical conditions of the task. Our findings provide insights into the behavioral mechanisms that allow for fast learning, suggesting a framework for rule learning as part of a multilevel learning scheme. We hypothesize that this framework will be useful for deciphering the neuronal mechanisms of rapid discrimination learning requiring reference memory.

## Introduction

Solving a discrimination task for a reward involves associative operant learning (1). Through experience, we learn the relationship between stimuli, actions, and results, aligning our actions with the desired outcomes. As we practice the task, certain aspects become easier and are eventually performed implicitly. Now consider a sudden change in some of the rules of a well-known task. The ability to adapt to new rules or learn from a small training set is a fundamental attribute of human cognition (2). However, it is not clear which behavioral patterns underlie adaptation, and what conditions allow fast and successful learning of new rules governing a well-known task.

In two-alternative forced-choice (2AFC) discrimination tasks, a discrimination criterion (DC) involves two stimulus–response associations. One way to learn a new DC governing a well-known discrimination task is to generalize from a known DC. Rodents, which provide a convenient model system for studying the neurobiological basis of behavior, can use generalization (3) and transfer (4, 5) to learn new rules. However, generalization cannot be used when a new DC is uncorrelated with previously learned DCs. A second option for successful performance when the DCs change is categorization, an established ability among rodents (6, 7). However, new criteria do not necessarily fall into previously acquainted categories. Third, higher levels of attention and experience may facilitate faster learning (8). Attending to details increases the learning rate (9). Furthermore, because learning is never carried out on a completely blank slate, previous knowledge (i.e. experience) may facilitate the acquisition of new DCs via learning sets (10) and schemas (11, 12). Indeed, during repeated changes to the DCs of a well-known task, experience may facilitate learning.

Although advantageous, extensive experience is not necessary for learning. Fast and even one-shot learning (13) were previously demonstrated in laboratory rodents. During fear conditioning, a stimulus is associated with a single exposure to an aversive experience, leading to avoidance learning (14, 15). Rodents also excel in spatial learning (16) and can learn from a few exposures (17, 18) or even from a single spatial experience (19–21). However, classical learning and operant learning are associated with distinct neuronal mechanisms (22, 23). Furthermore, although rodents are capable of quick learning of naturalistic tasks, many operant learning tasks are conducted on naïve laboratory subjects (12) and take weeks to learn (6, 24–28). Thus, it is unknown how rapidly can rodents learn a new set of associations within a well-known setting and what is the specific contribution of experience.

Rule learning in a discrimination task with changing DCs can be divided into four distinct levels of learning. The first level is a type of procedural learning, in which the general task logic is acquired, e.g. learning that there are water ports. The second level involves learning that there is a pair of stimulus–response associations that together constitute a DC. The third level involves learning that a specific pair of stimuli is now associated with a specific pair of responses. The fourth level is “learning to learn,” i.e. learning that the DC governing the task can change between days, and being able to adapt more quickly.

Using a fully automated 2AFC paradigm, we find that every mouse learns a new visual DC in a single session. When single session learning (SSL) occurs, mice perform the task successfully after being exposed to only three testing trials. Physically difficult DCs are less likely to result in SSL, and experienced mice are more likely to achieve SSL. SSL can be achieved for particularly difficult DCs if conditions for generalization from a previously learned DC are favorable. Thus, mice learn to learn.

## Results

### All mice learn at least one new visual DC within a single session

**Fig. 1.**
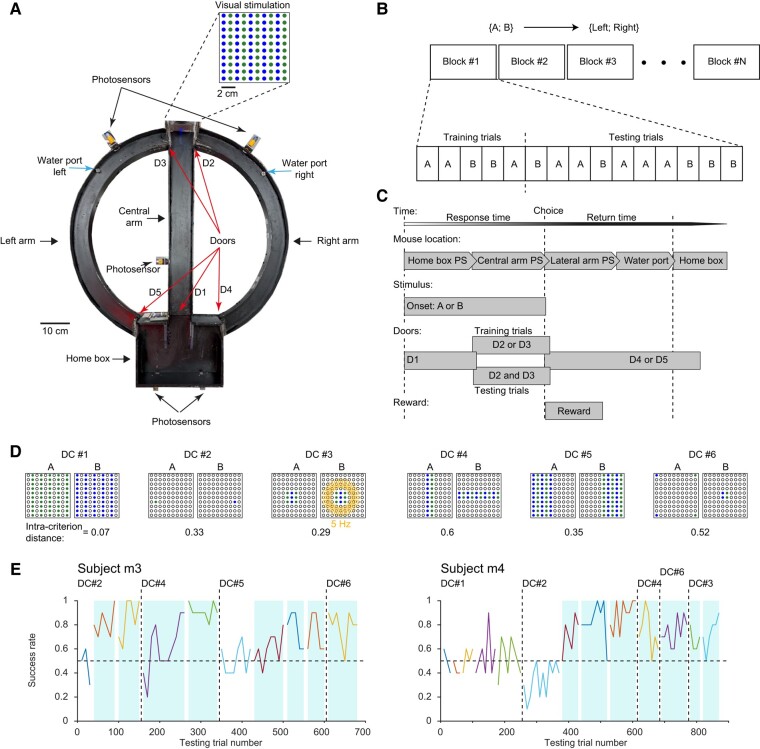
All mice learn at least one new visual DC within a single session. A) The fully automated apparatus used to train freely moving mice on a 2AFC discrimination task. Successful trials are reinforced by water. Top: The display used to generate visual DCs, located at the far end of the central arm, consists of 10 alternating columns of green and blue LEDs. B) Session structure. Every session is composed of blocks, each consisting of 5 training trials, followed by 10 testing trials. C) A trial structure. During training trials, only the correct choice is available to the animal since only one door at the T-junction is open (D2 or D3). D) Six visual DCs are used to govern the task. On every session, only one DC is used. Bottom: The physical properties of two visual stimuli {A; B} are used to derive an intracriterion distance metric, quantifying DC difficulty. E) Success rate as a function of testing trial number for two example subjects, m3 and m4. Sessions are represented by distinct colors, and the success rate is averaged per block. A blue background highlights successful sessions (*P* < 0.05, binomial test comparing to chance level, 0.5).

We designed a fully automated 2AFC discrimination paradigm for freely moving mice in a T-maze (Fig. 1A). All subjects were hybrid mice (21) (first-generation offspring of FVB/NJ females and C57BL/6J males; *n* = 6) that were pretrained (“shaped”) over several days (median [interquartile range, IQR]: 5 [4 5] days). To maximize learning rate and minimize frustration, every postshaping session was divided into blocks (Fig. 1B). Each block consisted of 5 training trials, followed by 10 testing trials. During training trials, the subjects were presented with the same stimuli as during testing trials, but the correct choice was enforced by opening only one door at the T-junction (Fig. 1C). Mice received a water reward after every training trial and every successful testing trial.

During every session, a display of 100 green and blue LEDs (Fig. 1A, inset) was used to provide 2 distinct visual stimuli {A; B} for governing the task. Thus, a DC was composed of two stimulus–response associations, an affinity between stimulus A and “go left” and an affinity between stimulus B and “go right.” Only one DC was employed during a given session, repeated across all blocks within that session. Every specific set of LEDs was included as a visual stimulus in only one DC. The physical properties of the two visual stimuli allowed characterizing every DC by an intracriterion distance in the range of [0, 1] and were smaller for more difficult DCs (Fig. 1D). For example, DC #1 consisted of {stimulus A: all green LEDs are on; stimulus B: all blue LEDs are on} and had an intracriterion distance of 0.07, indicating that the DC is relatively difficult.

Sessions were denoted as successful if performance was consistently above chance (0.5; *P* < 0.05, Binomial test). A DC was deemed successfully learned if a successful session was performed, while the task was governed by the DC. Using the 6-DC pool (Fig. 1D), 6 mice were tested on 28 DCs, with every subject exposed to a median [range] of 5 [3, 6] DCs. All subjects achieved an SSL of at least 1 DC (2 [1, 3]; Table 1 and Fig. S1A). When exposed to a new DC, a median [IQR] of 90 [70 138] testing trials were carried out until a successful session was completed (Fig. S1B). For example, after pretraining (shaping) on DC #2, subject m3 performed the task successfully during the second and third testing sessions (Fig. 1E, left). The criterion was then changed to DC #4, which was learned in a single session. Subject m3 was tested on three new DCs and achieved SSL on two. Similar results were obtained for all subjects (Table 1 and Fig. S1). Thus, all mice learned a least one new visual DC within a single session.

**Table 1.** SSL in every experimental animal.

No.	Animal ID	Sex	Strain^a^	Age^b^ (week)	Weight^b^ (g)	Nonfirst DCs^c^	SSL DCs
m1	mC41	Male	Hybrid	26	39.4	4	1
m2	mA154	Female	Hybrid	14	26.5	2	2
m3	mA350	Female	Hybrid	13	15.7	3	2
m4	mA354	Female	Hybrid	13	16.4	4	3
m5	mE177	Female	Hybrid	21	22.4	5	3
m6	mE178	Female	Hybrid	21	26.3	4	2
Total						22	13

^a^F_1_ offspring of an FVB/NJ female (JAX #001800) and a C57-derived male.

^b^At the beginning of training.

^c^Before being exposed to a new rule, every subject was shaped on one other DC.

### During SSL, mice achieve high success rates from the first trials of the first block

To determine the learning dynamics of SSL, we classified sessions according to the learning process by characterizing the success rate as a function of block number. Each session was associated with either SSL (e.g. m4, DC #6; Figs. 1E, right, and 2A), multisession learning (MSL; e.g. m4, DC #2; Figs. 1E, right, and 2B), or was unlabeled (e.g. m3, DC #2; Fig. 1E, left). By definition, a DC associated with MSL requires more than one session to learn. Since every mouse was pretrained on the first DC encountered by the subject, all first-DC sessions were unlabeled. Of the newly encountered DCs, 13/22 (59%) were SSL, 8/22 (36%; spanning 19 sessions) were MSL, and in one case, the DC was not learned (1/22, 5%; m5, DC #1; Fig. S1C and D). Thus, in 95% of the cases, mice learned a completely new DC within one to three sessions.

**Fig. 2.**
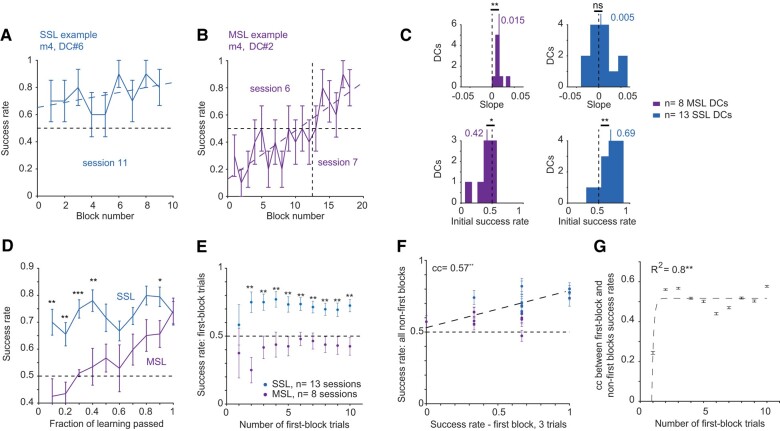
During SSL, mice achieve high success rates from the first trials of the first block. A) Example SSL curve. In A and B, the dashed lines represent linear model fit. In A, B, D, E, and F, the error bars represent SEM. B) Example MSL curve: a linear model fitted to the combined two sessions. C) A linear model was fit to every learning curve. Top: slopes. MSL but not SSL curves have slopes consistently above zero. Bottom: intercepts. SSL but not MSL curves have initial success rates consistently above chance. ns, *P* > 0.05; **P < 0.01; Wilcoxon's test comparing to chance level. D) The success rate of SSL is consistently higher than that of MSL during the initial 40% of the session. In D and E, *P < 0.05, **P < 0.01, ***P < 0.001, *U* test. E) Success rates of the first 1, 2, 3, …, or 10 testing trials of SSL and MSL sessions. F) Success rates in all nonfirst blocks of the first sessions of newly encountered DCs vs. success rates in the first three trials of the session. Dashed line, linear fit; cc, rank correlation coefficient; **P < 0.01, permutation test. G) cc-s between the success rate during the first few first-block testing trials and all nonfirst blocks, plotted against the number of first-block testing trials. Dashed line, exponential fit; **P < 0.01; *F* test. Error bars, SD. The correlation stabilizes after three testing trials.

SSL success rates (0.7 [0.6 0.8]; *n* = 110 blocks) were higher than MSL success rates (0.6 [0.5 0.7]; *n* = 154; *P* < 0.001, *U* test; Fig. S1E). To quantify differences in the learning process, a linear model was fit to every SSL and MSL learning curve (Fig. 2A and B, dashed lines). During MSL, mice exhibited gradually increasing success rates (median slope: 0.015 improvement per block; *n* = 8 MSL DCs; *P* = 0.008, Wilcoxon's test comparing to a zero-slope null; Fig. 2C, top left), indicating a learning process. MSL initial success rates were below chance (0.5; median: 0.42; *P* = 0.039; Fig. 2C, bottom left). In contrast, SSL initial success rates were already above chance (0.69; *n* = 13; *P* = 0.003) and did not increase consistently over blocks (median slope: 0.005; *P* = 0.84; Fig. 2C, right). Thus, when SSL occurred, the trials experienced during the first block appeared to suffice for learning the new DC.

Linear models do not necessarily capture differences between learning curves. Instead of considering models that require more free parameters (e.g. sigmoid), we time-warped every curve to unity duration and averaged all learning curves of a given type (SSL or MSL; Fig. 2D). SSL and MSL success rates during the initial four tenths were higher for SSL sessions than for MSL sessions (geometric mean, *P* = 0.003, *U* test; Fig. 2D). These observations complement the linear model results, showing that during SSL, animals learn the new DC during the very first block.

To understand how subjects can perform above chance from the first block in a new DC session, we considered two alternatives. One possibility is that the mice already learn the new DC during the five training trials (Fig. 1B) at the beginning of the first block. Alternatively, one or more testing trials are required. We found that success rates during the first two (or more) testing trials of a new SSL DC were consistently higher than the success rates of first trials in an MSL DC (*P* < 0.01 in all cases; *n* = 13 SSL and 8 MSL first-DC sessions; Fig. 2E). To estimate the number of testing trials actually required for learning a new DC, we calculated the correlation between the success rate during the first few testing trials of the first new-DC block and the success rate during all other same-session blocks (Fig. 2F and G). There was no consistent correlation when a single first-block trial was considered (cc: 0.24; *n* = 21 sessions; *P* = 0.33, permutation test). However, when two or more first-block trials were considered, the correlation was high (range, [0.44, 0.58]; *P* < 0.05 in all cases, permutation tests; Fig. 2F and G). An exponential model provided a fit to the correlation as a function of the number of first-block trials (*R*^2^ = 0.8; *n* = 10; *P* = 0.003 *F* test; Fig. 2G), indicating that the correlation already converged for three testing trials (Fig. 2F). Thus, after the five training trials, the first three testing trials exhibited the same correlation as the entire first block with the session success rate, indicating that when the conditions are appropriate, three testing trials suffice for learning a new DC.

### SSL is predicted from experience and criterion difficulty

**Fig. 3.**
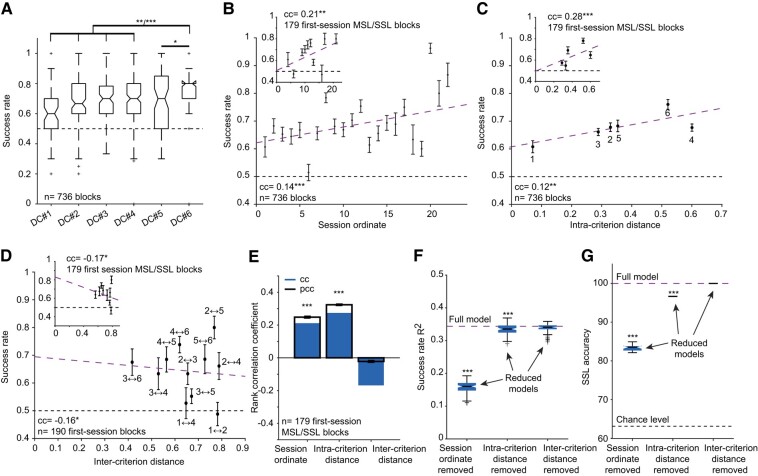
SSL is predicted from experience and rule difficulty. A) Success rates for individual DCs. *P < 0.05, **P < 0.01, ***P < 0.001, Kruskal–Wallis test, corrected for multiple comparisons. Every box plot shows median and IQR, whiskers extend for 1.5 times the IQR in every direction, and a plus indicates an outlier. B) Success rates vs. animal experience, quantified by the session ordinate. In B–D, purple lines, linear model fit; error bars, SEM. Inset: success rate vs. session ordinate for a reduced set of SSL and MSL sessions. In B–E, *P < 0.05, **P < 0.01, ***P < 0.001, permutation test. C) Success rates vs. DC difficulty, quantified by the intracriterion distance metric (Fig. 1D). D) Success rates vs. the similarity between the new and the previous DC. Only first posttransition sessions are included. E) cc-s and partial rank correlation coefficients (pcc) between success rate and the features described in B–D. Error bars, SD. F) Variance in block success rate (*R*^2^) explained by cross-validated support vector regression models. ***P < 0.001, Bonferroni-corrected *U* test between the *R*^2^ of the full model and the *R*^2^ of the reduced model obtained after removing one feature. G) Accuracy in predicting SSL using cross-validated support vector classification. ***P < 0.001, Bonferroni-corrected *U* test between the accuracy of the full and the reduced models.

What physical and mental conditions are appropriate for SSL? To assess what determines SSL, we first examined how criterion identity affects success rates. Mice exhibited different success rates on different DCs (Fig. 3A). For instance, DC #6 (Fig. 1D) was associated with the highest success (median [IQR]: 0.8 [0.7 0.9]; *n* = 65 blocks; geometric mean of five comparisons, *P* = 0.00007, Kruskal–Wallis test; Fig. 3A). In contrast, DC #1 was associated with the lowest success rates (0.6 [0.5–0.7]; *n* = 67; *P* = 0.006; Fig. 3A). Thus, success rates depend on the specific criterion.

When a new session starts and the DC is replaced, mice are compelled to learn the new associations quickly to maximize the reward, requiring memorization of the outcomes of previous trials during the same session (reference memory (29–32)). However, other aspects of the task may be learned gradually over multiple sessions. We found that success rates correlated with the accumulated experience of the animal, quantified by the session ordinate (number of previous sessions; cc: 0.14; *n* = 736 blocks; *P* < 0.001, permutation test; Fig. 3B). The correlation was not consistently different (*P* = 0.22, bootstrap test) when only the first sessions involving SSL and MSL were included (cc: 0.21; *n* = 179 first-session blocks; *P* = 0.0043; Fig. 3B, inset). Thus, when mice are more experienced, performance is better when encountering a new DC.

Even if experience contributes to successful performance, DC difficulty may influence the success rate. However, it is not a priori clear what a mouse considers “difficult.” We quantified DC difficulty using the intracriterion distance metric based on the physical properties of the stimuli (Materials and Methods; Fig. 1D). Success rates correlated with intra-DC distance (cc: 0.12; *n* = 736 blocks; *P* = 0.002, permutation test; Fig. 3C). A higher correlation (*P* = 0.014, bootstrap test) was observed when only the first SSL and MSL sessions were considered (cc: 0.28; *n* = 179 blocks; *P* < 0.001; Fig. 3C, inset). Thus, success rates are correlated with intracriterion distance, especially when a new DC is encountered.

Another possible route to successful performance is generalization from a previously learned DC. If mice generalize, a higher similarity between consecutively presented DCs may yield better performance, whereas grossly distinct DCs may induce confusion. We quantified the similarity between every 2 DCs using the intercriterion distance metric, which is near 0 when DCs are very similar and near 1 when DCs are very different from one another. In first-session blocks, the success rate was anticorrelated with intercriterion distance (cc: −0.16; *n* = 190 first-session blocks; *P* = 0.03, permutation test; Fig. 3D). Similar results (*P* = 0.48, bootstrap test) were obtained when only MSL and SSL first-session blocks were considered (cc: −0.17; *P* = 0.023; *n* = 179, i.e. without the 11 blocks of the never-learned DC #1 in m5; Fig. 3D, inset). Thus, when the new DC is more similar to the previously learned DC, success during the first session is already higher.

Based on the foregoing, success level depends on several intercorrelated features (Fig. S2B), including session ordinate (Fig. 3B), DC difficulty (Fig. 3C), intercriterion similarity (Fig. 3D), and the success during the initial testing trials (Fig. 2E–G). First, we used correlation analysis to disambiguate the features. When considering the first three features, the partial rank correlation coefficient (pcc) was consistently distinct from zero for the session ordinate (pcc: 0.25; *n* = 179 first-session blocks during MSL and SSL; *P* < 0.001, permutation test; Fig. 3E) and for intracriterion distance (pcc: 0.32; *P* < 0.001), but not for intercriterion distance (pcc: −0.02; *P* = 0.77). Similar results were obtained when success in the first three first-block trials was used as a fourth feature (Fig. S2C).

Second, to determine the total variability of the success rate explained by the three features, we used cross-validated support vector regression. Over a third of the variability in block success rate was explained (*R*^2^ = 0.34 [0.33 0.35]; 179 blocks; median [IQR] of *n* = 20 independent 10-fold cross-validated models; Fig. 3F, Full model). By excluding one feature at a time, we found that the session ordinate made the dominant contribution to success rate (*R*^2^ = 0.16; *P* < 0.001, *U* test corrected for multiple comparisons; Fig. 3F, Reduced models). Intracriterion distance also made a consistent contribution (*R*^2^ = 0.33; *P* < 0.001), whereas intercriterion distance did not (*R*^2^ = 0.34, *P* = 0.06). Similar results were observed when success in the first three first-block trials was included (Fig. S2D). Thus, the most important single feature for determining the success rate during the first session of a new DC is the accumulated experience.

Knowing that success rate during the first session of a newly encountered DC depends on the accumulated experience and DC difficulty, we assessed what determines SSL. We used cross-validated binary classification (support vector machines) to predict whether a given first session block is SSL or MSL (*n* = 179 blocks). The prediction of SSL from animal experience and physical DC properties was perfect (100% accuracy; Fig. 3G, Full model). By again excluding one feature at a time, we found that prediction depended on session ordinate (experience; 83% [83 84%]; *P* < 0.001, *U* test; Fig. 3G, Reduced models) and on intracriterion distance (DC difficulty; 97% [97 97%]; *P* < 0.001). Prediction did not depend on intercriterion distance (*P* = 1). A priori, it is possible that if the animal randomly guesses the correct response in the first testing trials, SSL is more likely to be achieved. However, we found that when success in the first three first-block trials was considered, similar results were obtained, with the session ordinate making the dominant contribution in all cases (Fig. S2E and F). Thus, knowing how experienced an animal is and what the physical properties of a new DC are allows for predicting whether SSL will occur.

### When conditions are favorable, mice can generalize from similar yet easier DCs

**Fig. 4.**
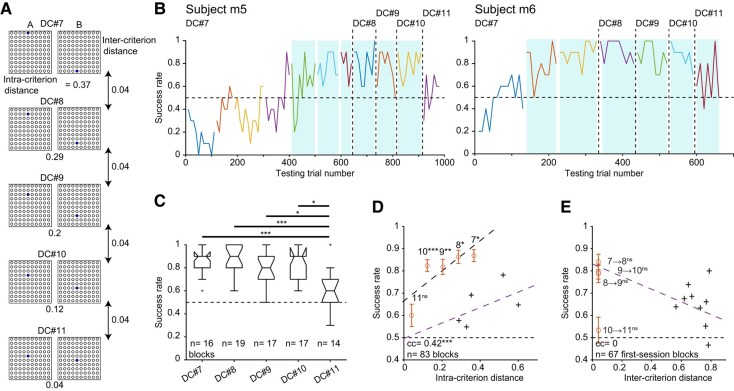
When conditions are favorable, mice can generalize from similar yet easier DCs. A) Five visual DCs of increasing difficulty distinct from DCs #1–6 (Fig. 1D) used consecutively in subjects m5 and m6. B) Success rates as a function of testing trial number. After performance on DC #7 stabilized, a new DC of higher difficulty was employed on every session. All conventions are the same as in Fig. 1E. C) Success rates for individual DCs. In C–E, only the last session of DC #7 was used. *P < 0.05, ***P < 0.001, Kruskal–Wallis test. D) Success rate vs. DC difficulty. ***P < 0.001, permutation test. In D and E, crosses and purple lines represent data points and linear models copied from Fig. 3C, inset (and from Fig. 3D, inset). In D and E, ns, *P* > 0.05; *P < 0.05, **P < 0.01, ***P < 0.001, *U* test; error bars, SEM. E) Success rate vs. the similarity between the new and the previous DC.

The negligible reduction in variability explained by intercriterion similarity during SSL and MSL sessions (Figs. 3F and S2D) suggests that mouse strategy was not based on generalization or categorization according to the previously learned DC. However, minimizing the usage of generalization may be specific to the set of arbitrary DCs (Fig. 1D), for which median [IQR] intercriterion distances were 0.65 [0.53 0.77] (*n* = 11 transitions; Fig. 3D). To determine whether mice employ a different strategy when consecutive DCs are similar, we tested two of the subjects on a set of five new nonarbitrary DCs (m5 and m6; Fig. 4). The first new DC was the easiest (highest intracriterion distance), followed by gradually more difficult DCs (Fig. 4A). The intercriterion distance between every pair of consecutively presented DCs was fixed at 0.04. To minimize the direct effect of the most recently learned arbitrary DCs, animals were kept away from the apparatus for 7 days before being presented with the first new DC, and no pretraining (shaping) sessions were conducted. After reaching stable performance on the first new DC (#7), every other DC was presented for one session only. None of the mice managed to learn the first DC during the first session. However, despite the increasing difficulty, both mice achieved SSL for DCs #8–10 (Fig. 4B). For instance, intracriterion distances of DC #9 and DC #10 were 0.2 and 0.12 (Fig. 4A), more difficult than DCs #2–6 that were associated with SSL (range: [0.29, 0.6]; Fig. 1D). Nevertheless, SSL was readily achieved for DCs #8–10. Even the most difficult DC (#11; intracriterion distance, 0.04) was associated with SSL in one of the two subjects (m6; Fig. 4B). Thus, when intercriterion similarity is high, SSL can be achieved even for difficult DCs.

The set of new DCs #7–11 was presented in the same order to both mice. However, success rates during DCs #8–10 were not consistently lower than during the last session of DC #7 (*n* = 19, *n* = 17, and *n* = 17 blocks; *P* = 1, *P* = 0.86, and *P* = 0.85, Kruskal–Wallis test; Fig. 4C). In contrast, success rates during DC #11 were lower than during DC #10 (*P* = 0.02), although the intercriterion distance between DC #10 and DC #11 was identical to the distance between every other pair of consecutively presented DCs. Success rates were correlated with intracriterion distance (cc: 0.42; *n* = 83 blocks; *P* < 0.001, permutation test; black dashed line in Fig. 4D). However, the distances between the points representing DCs #7–10 and the linear fit to the arbitrary DCs (Fig. 4D, purple line) were higher than for DCs #1–6 (*n* = 19, *n* = 17, *n* = 17, and *n* = 14 blocks; *P* = 0.013, *P* = 0.0013, *P* = 0.0013, and *P* = 0.0004, *U* test; Fig. 4D). In contrast, DC #11 did not deviate from the line (*n* = 14 blocks; *P* = 1), suggesting that under the conditions of the task, DC #11 was close to the “just noticeable difference” of visual discrimination. Regardless, the success rates of DCs #7–10 are higher than expected given the difficulty of the DCs and the pattern established by the arbitrary DCs (Fig. 3), indicating a strategy shift.

The higher than expected success rates of DCs #8–10 may be explained by generalization from similar yet easier DCs—or by categorizing the new samples according to some overarching criterion, e.g. above/below the horizon. Since all intercriterion distances were identical (0.04), success rates could not be directly correlated with intercriterion distances (Fig. 4E). The distances of DCs #8–11 from the linear fit to the arbitrary DCs were not consistently different than for DCs #1–6 (*P* = 0.6, *P* = 0.62, *P* = 0.11, and *P* = 0.056, *U* test; Fig. 4E). Thus, SSL of particularly difficult DCs can be facilitated by generalization from similar yet easier DCs. Indeed, success rates during SSL sessions with low intercriterion distances (DCs #7–11; 0.85 [0.7–0.9]; *n* = 67 blocks) were consistently higher than for high intercriterion distances (DCs #1–6; 0.7 [0.6 0.8], *n* = 112; *P* = 0.0002, *U* test). In sum, when conditions for generalization are more favorable, mice perform SSL with a higher success rate.

## Discussion

We tested the ability of mice to learn new DCs that span a range of difficulties and quantified the requisite conditions. After acquiring the basic paradigm, subjects gradually improve their performance on a new visual DC, and eventually learn a new DC within a single session. Remarkably, all animals achieve SSL of at least one DC. When SSL occurs, mice successfully perform the task after being exposed to five training and three testing trials. SSL is achieved when mice are experienced, when the DC is relatively easy, or when consecutive DCs are similar.

### Learning a DC

We defined a DC using a pair of stimulus–response associations. In a 2AFC paradigm, the subject can achieve perfect success without learning both associations. When only a single association is learned, solving the task still requires discrimination between the two stimuli. Thus, there are three possibilities for learning the DC: learn both associations; learn one association; or learn the other association. Yet in all three cases, the discrimination between the two stimuli must be achieved.

### Framework for rule learning as part of multilevel learning

Rule learning in a discrimination task with changing DCs can be divided into four distinct levels of learning. The first is a type of procedural learning, in which the general task logic is acquired. In the present 2AFC paradigm, the logic involved learning that certain running directions are permitted, that doors open and close, that water is available at two specific locations, and so on. The logic is learned during the shaping period (in the days prior to the first testing session), is independent of the existence of any specific DC, and remains relevant for all DCs.

The second level involves learning that there is a stimulus–response contingency (e.g. stimuli {A; B} are associated with {go left; go right} responses). Here, the second level involved learning that during testing trials, water is available only if specific stimulus–response contingencies are met. The first two levels of learning generate the long-term memory component of standard successive conditional discrimination tasks (24). When shaping is conducted prior to training on a specific stimulus–response contingency, the two levels may be separated (6, 33). Then, performance is tested on the second level.

The third level involves learning a specific new DC in a well-known setting and is only rarely assessed in animal studies (but see Refs. (4, 11, 12)). Successful performance may be achieved by generalization or transfer from a similar rule (4, 5), by applying a familiar rule to a new set of stimuli (categorization (6, 34, 35)), or by associative learning of the new contingencies (the present work). By definition, learning a new DC during a single session requires reference memory (29–32). If a new DC is not introduced, the third level cannot be assessed.

The fourth level, learning that the DCs of the task can change between sessions, is a long-term memory component that improves with experience and is independent of the first two levels of learning. When the DC is fixed over many sessions, the subject may still improve between sessions. However, the ability of learning to learn a new DC can be assessed only when the DCs change. In the present work, we focused on the third and fourth levels.

Tasks that involve generalization (3, 34, 36), transfer (4, 5), or categorization (6, 34, 35) involve the first two levels but do not require associative learning. The finding that in experienced mice, a new DC can be learned by the end of the first three testing trials indicates that mice can learn to discriminate from a small number of samples, emphasizing the importance of reference memory when conditions in a familiar environment change. Previous work showed that rodents can learn from a few samples in various settings including the Morris water maze (19, 20), fear conditioning (14, 15), or labyrinth navigation (17). Yet in all the aforementioned studies, only two levels of learning (procedural and stimulus–response contingency) were tested. In the radial arm maze (29), the specific set of arms baited during the session allows to also test the third level (reference memory). However, the rule governing the task remains unchanged. Thus, though previously addressed (10, 12, 37), understanding of the process of learning to learn in animal subjects is still limited.

The finding that experience is crucial for SSL becomes clear considering the multilevel learning framework suggested here. The learning-to-learn process (fourth level) implies that a more experienced animal is more flexible to changes in the environment and more likely to learn a new DC quickly. Nevertheless, learning a DC during SSL is by definition limited to a single session. Thus, learning a specific new DC (the third level) is a process distinct from all processes that depend on long-term memory. The fact that easier DCs are more likely to be learned suggests a dissociation between the third and fourth levels, supporting the multilevel framework.

### Generalization and categorization

Other cognitive processes may be utilized when two consecutive DCs are similar. Learning a new DC when the conditions for generalization are favorable is not independent of the past. Previous work found that rats can use transfer learning to perform a difficult discrimination task (4). Rodents also excel in categorization (6, 38, 39). In both cases, the animal generalizes from previously learned DCs and can solve the task without learning new associations, as in the fourth level of learning suggested above. Indeed, when intercriterion distances were lower, success rates were higher. Furthermore, SSL was achieved even for difficult new DCs, suggesting that mice did not necessarily learn the new DC, which would have required associative learning and reference memory. Instead, the animals may have categorized the stimuli comprising the new DC using previously acquired knowledge.

### Flexibility across learning organisms and systems

Cognitive flexibility (40, 41) is the capacity to alternate between two distinct concepts based on the context. A related concept, behavioral flexibility (42), refers to the modification of behavior in reaction to shifts in environmental circumstances. With humans, the Wisconsin Card Sorting Test (WCST (43, 44)) and the intradimensional/extradimensional test (ID/ED (45)) have been used to assess flexibility. The WCST has also been used to study cognitive flexibility in primates (46–48), while ID/ED testing can be used with rodents (49). Complementing the neuropsychological tests for flexibility, the computer science field of meta-learning (50–53) involves algorithms that learn from diverse tasks and leverage accumulated experience to rapidly adapt to new tasks. The mouse paradigm we employed requires capability for cognitive flexibility, akin to the WCST and ID/ED tasks, while allowing responses that capitalize on the natural spatial exploration tendency of mice. We suggest that the present paradigm could be used to test models of cognitive impairment in rodents.

### Limitations

A couple of limitations should be noted. First, while intracriterion distances spanned the entire range of possible values, intercriterion distances were sampled only for high (>0.4) or very low (0.04) values. When encountering intermediate intercriterion distances, mice may exhibit yet a third strategy, or perhaps change their strategy dynamically according to the relation between intra- and intercriterion distances. Second, no fixed mathematical transformation couples the two stimuli that formed a DC, and every visual stimulus was used in only one DC. Our choice to employ new stimuli in every DC while keeping the responses fixed resulted from practical considerations. Specifically, the two-arm maze provides two diametrically opposing natural responses, whereas swapping stimulus–response associations involves reversal learning—which we wanted to avoid. Other possible implementations could involve changing only one stimulus in a DC, swapping the associations between the two stimuli and responses that constitute a DC, or associating new responses with familiar stimuli.

### A neural hypothesis for rule learning

Due to high reproductive rate, genetic control, and well-established tasks for a plethora of behaviors, mice have emerged as a robust model for studying various neuronal mechanisms (54–58). Head-fixed and freely moving mice allow combining behavioral and neuronal recordings (24, 26) and manipulations (57–60). Extensive correlative evidence links neuronal activity with learning, perception, and discrimination (61–63). Indeed, neuronal mechanisms underlying operant learning are often studied using rodents performing a sensory discrimination task (26, 63–67). The mechanisms that underlie learning in a multirule environment have been studied from theoretical and empirical perspectives (68, 69). However, learning across multiple sessions limits the interpretational power yielded by many rodent learning tasks because in many cases, the same neuronal population cannot be guaranteed to be followed over long durations. Thus, we expect that SSL will facilitate studying the neurophysiological basis of learning by increasing the overlap between the recorded neuronal activity and the act of learning.

The cellular-network mechanisms underlying the process of rapid discrimination learning requiring the use of reference memory are presently unknown. We hypothesize that the framework of multilevel learning will be useful for deciphering these mechanisms. Moreover, the mechanisms that underlie the enhancement of learning capabilities are unknown. We hypothesize that the timescale of synaptic transmission, the precision of synaptic transmission, the signal-to-noise ratio in neuronal information processing, and plasticity timescales are all modified as a subject becomes a better learner.

## Materials and methods

### Experimental animals

A total of six adult hybrid mice were used in this study, one male and five females (Table 1). Hybrid mice were used since compared to the progenitors, hybrids exhibit reduced anxiety-like behavior, improved learning, and enhanced running behavior (21). Four of the mice (m1–m4) were hybrid and double transgenic, the F_1_ generation of an FVB/NJ female (JAX #001800, The Jackson Labs) and a male offspring of an Ai32 female (JAX #012569) and a CaMKII-Cre male (JAX #005359). The other two mice (m5 and m6) were offspring of an FVB/NJ female and a PV-Cre male (JAX #008069). In two subjects (m1 and m4), electrophysiological recordings and optical manipulations were carried out during some sessions. The results of electrophysiological recordings and optogenetic manipulations are not included in the present report. All behavioral results were observed at the subject level (Table 1 and Fig. S1) and no differences were observed between implanted and un-implanted subjects. After separation from the parents, animals were housed in groups of same-litter siblings until participation in experiments. Animals were held on a reverse dark/light cycle (dark phase, from 8 AM to 8 PM). All animal handling procedures were in accordance with Directive 2010/63/EU of the European Parliament, complied with Israeli Animal Welfare Law (1994), and were approved by the Tel Aviv University Institutional Animal Care and Use Committee (IACUC #01-16-051 and #01-21-061).

### Water deprivation

Mice were trained on a 2AFC task in which the DCs governing discrimination behavior could change between different sessions. Every session was conducted on a different day. At the beginning of the training period, animals were housed one per cage and placed on a water-restriction schedule that guaranteed at least 40 mL/kg of water every day, corresponding to 1 mL for a 25-g mouse. Training was carried out 5 days a week, and animals received free water on the sixth day. Reward volume differed between mice and sessions, ranging 4–20 µL. The exact volume was determined by the experimenter before each session based on familiarity with the specific animal. In all sessions, the reward was larger by 20–50% during testing compared with training trials.

### Apparatus

The apparatus was a circular T-maze equipped with 5 motorized doors, 5 photosensors, 2 solenoid-driven reward ports, and a 100-LED visual stimulation matrix (Fig. 1A). All sensors and actuators were controlled by a microcontroller (Arduino Mega) via custom designed electronic circuitry. The home box (L × W × H: 20 × 30 × 10 cm) was located at the beginning of the central arm (75 × 8 × 3 cm) and connected to the end of the two lateral arms (100 × 6 × 3 cm). Each passageway between the home box and one of the arms was blocked by a transparent polycarbonate door. Two additional doors were located at the sides of the T-junction at the end of the central arm, blocking passage to the lateral arms. Every door was operated by a small motor (DC 6V 30RPM Gear Motor, Uxcell) and was equipped with two limit switches (D2F-01L2, Omron). There were five photosensors (S51-PA-2-A00-NK, Datasensor). Water rewards were given by solenoids (003-0137-900, Parker), and each water port was connected to a different solenoid via flexible (Tygon) tubing. The visual stimulation matrix was constructed of 10 × 10 LEDs in alternating columns of blue (470 nm, Cree) and green (527 nm, Cree) diodes.

### Discrimination task

On a given session, a single DC, including two associations, was used (Fig. 1D). The allocation of DCs to sessions was pseudo-random. Stimulus A was associated with leftward runs, and stimulus B was associated with rightward runs. The allocation of stimuli A and B to trials was pseudo-random. There were two types of sessions, “shaping” (pretraining) and “learning.” Shaping sessions included only “training” trials. Initially, each mouse was acquainted with the task in a series of shaping sessions (median [range]: 5 [4, 10] sessions). Mice had to reach a criterion of 50 trials per session before commencing learning sessions. On each session, mice were free to perform the task until losing interest. Loss of interest was identified by behavior that included prolonged periods of rest and attempts to climb the walls of the home box. Learning sessions were divided into blocks (Fig. 1B), and every block included 5 training trials and 10 “testing” trials.

A single testing trial proceeded as follows: (i) *Stimulus*: Once the animal entered the home box and passed the photosensors, the entrance doors (D4 and D5) closed, and the exit door to the central arm (D1) opened (Fig. 1A). At the same time, a stimulus A or B was given, remaining available to the animal until a lateral photosensor was passed during the response. (ii) *Run*: Once the animal left the home box and passed the central arm photosensor, the two lateral doors (D2 and D3) at the T-junction opened, and the exit door (D1) closed. During the Run, the stimulus was continuously available. (iii) *Response*: The animal chose a direction at the T-junction and went through one of the two open doors. Once the animal passed the lateral arm photosensor, the stimulus turned off and the lateral doors closed, preventing the animal from changing the choice. (iv) *Reward*: A liquid reward was available at the corresponding water port if the animal made a correct choice. The animal could not go back to the T-junction, but was free to consume the reward and return to the home box through the open home box door (D4 or D5).

During training trials, only the corresponding door (D2 or D3) at the T-junction opened. Because the animal could not make an incorrect response, a reward was given during every training trial.

### Quantification of criterion difficulty and intercriterion similarity

To place arbitrary DCs on a continuous scale, a symmetric intracriterion distance metric was introduced. Each stimulus was represented as a 100-element binary 2D array, with each element taking the value of 0 or 1 indicating whether the corresponding LED was off or on. The chromatic component (LED color) was ignored. To derive the metric, every DC was characterized by three physical attributes that quantified the difference between the stimuli A and B. (i) The correlation distance (cd): one minus the maximal value of the 2D cross-correlation coefficient between A and B. (ii) The Euclidean distance (ed): the scaled distance between the optimal match that yields the correlation distance. (iii) The luminance distance (ld): the scaled difference in luminance between A and B. All distances (cd, ed, and ld) are nonnegative scalars limited to the [0, 1] range. The distance metric was then the magnitude of the 3D vector, defined as


$$d_{\mathrm{intra}}=d_{\mathrm{AB}}=\sqrt{\frac{\mathrm{cd}{\left(A,B\right)}^{2}+\mathrm{ed}{\left(A,B\right)}^{2}+\mathrm{ld}{\left(A,B\right)}^{2}}{3}.}$$

The metric ranges [0, 1], taking the value of 0 when the two stimuli are identical (an impossibly difficult DC) and 1 when stimuli are maximally distinct (a very easy DC).

The same physical attributes that characterize each DC were used to measure the difference between distinct DCs (e.g. Fig. 4D). The intercriterion distance is a symmetric measure of the difference between the physical properties of the same-laterality stimuli of the two DCs. Thus, for a pair of DCs {A; B} and {A′; B′}, the intercriterion distance is the average of the intracriterion distances for two “mixed” DCs: {A; A′} and {B; B′}. The resulting metric is


$$d_{\mathrm{inter}}=(d_{AA^{\prime }}+d_{BB^{\prime }})/2.$$

The metric takes the value of 0 when the two DCs are identical and 1 when the DCs are maximally distinct. DC difficulty and intercriterion difficulty were calculated based on the physical properties of the stimuli themselves. Dynamic aspects, including viewing angle, distance, and retinal image, were ignored.

### Comparison of rank correlations

To determine whether there is a difference between two rank correlation coefficients (cc_1_ and cc_2_), we bootstrapped (resampling with replacement) the two datasets (*n* = 1,000 iterations) and calculated $c{c^{\prime }}_{1}$ and $c{c^{\prime }}_{2}$ for each iteration. We then calculated the difference between the pairs of $c{c^{\prime }}_{1}$ and $c{c^{\prime }}_{2}$ and quantified the probability (two-tailed) that the difference differs from zero.

### Statistical analyses

In all statistical tests used in this study, a significance threshold of *α* = 0.05 was used. All descriptive statistics (*n*, median, IQR, range, mean, SD, and SEM) can be found in the text, figures, and figure legends. All analyses were conducted in MATLAB (MathWorks). Nonparametric statistical tests were used throughout. Differences between the medians of two groups were examined using Mann–Whitney *U* test (two-tailed). Differences between the medians of three or more groups were tested with Kruskal–Wallis nonparametric analysis of variance and corrected for multiple comparisons using Tukey's procedure. Wilcoxon's signed-rank test was employed to determine whether a group median was distinct from zero (two-tailed). To estimate whether a given fraction was smaller or larger than expected by chance, an exact binomial test was used. A permutation test (70) was used to estimate the significance of rank correlation coefficients (cc and pcc).

## Supplementary Material

pgae203_Supplementary_Data

## Data Availability

The code used in this study is publicly available via GitHub (https://github.com/EranStarkLab/LearningToLearn). All data are publicly available via Zenodo (https://zenodo.org/records/11305363).

## Preprints

This manuscript was posted on the bioRxiv preprint server at the following address: https://www.biorxiv.org/content/10.1101/2023.03.28.534599.
