# Supplementary material for: Learning to learn: Single session acquisition of new rules by freely moving mice
Source: PNAS Nexus. 2024 May 19;3(5):pgae203. doi: 10.1093/pnasnexus/pgae203 (PMC11138122; doi:10.1093/pnasnexus/pgae203)
Supplement: pgae203_Supplementary_Data [file pgae203_supplementary_data.pdf]

Supplementary Information for

Learning to learn: Single session acquisition of new rules by freely-moving mice

Amir Levi[1,2,#], Noam Aviv[1], and Eran Stark[1,2,3,*]

[1]Department of Physiology and Pharmacology, Faculty of Medicine, Tel Aviv University, Tel Aviv 6997801, Israel
[2]Sagol School of Neuroscience, Tel Aviv University, Tel Aviv 6997801, Israel
[3]Sagol Department of Neurobiology, Haifa University, Haifa 3103301, Israel
[#]Present address: Department of Physiology, University of California, San Francisco, San Francisco, CA 94143, USA

Email: eranstark@sci.haifa.ac.il

This PDF file includes:

Figures S1 to S2

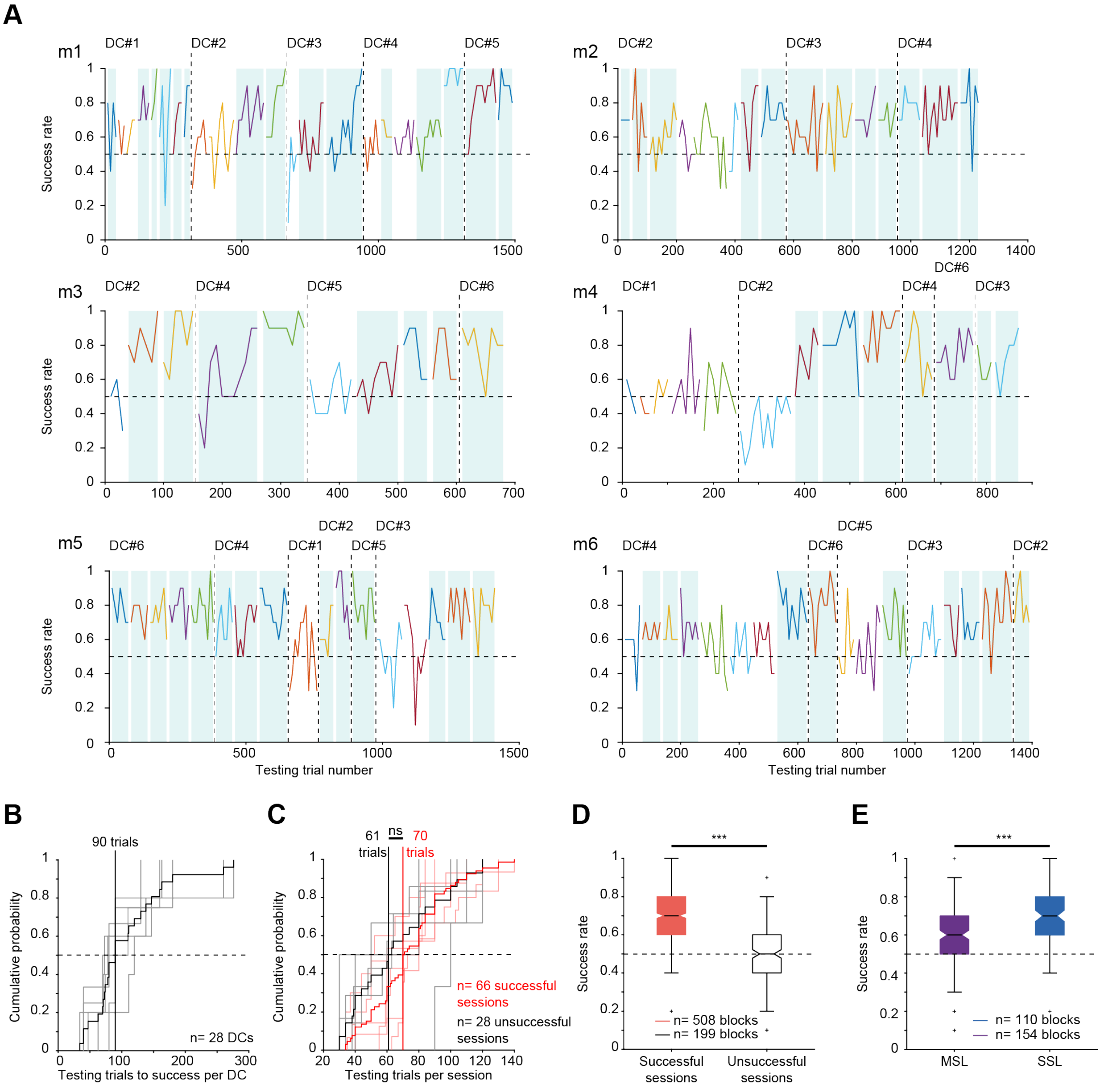

Figure S1. All mice achieve single session learning of at least one DC. (**A**) Every panel shows the success rate as a function of testing trial number for one subject. Sessions are represented by distinct colours, and every point indicates single-block success rate. A blue background highlights successful sessions (p<0.05, Binomial test comparing to chance level, 0.5). (**B**) Mice learn a new DC within a median of 90 testing trials. Data are from six mice tested on 28 DCs. Grey lines, individual mice. (**C**) The number of testing trials performed during successful and unsuccessful sessions are not consistently different. Here and in **DE**, ns/***: p>0.05/p<0.001, U-test. (**D**) Success rates are higher during successful compared with unsuccessful sessions. (**E**) Success rates are higher d SSL compared with MSL DCs.

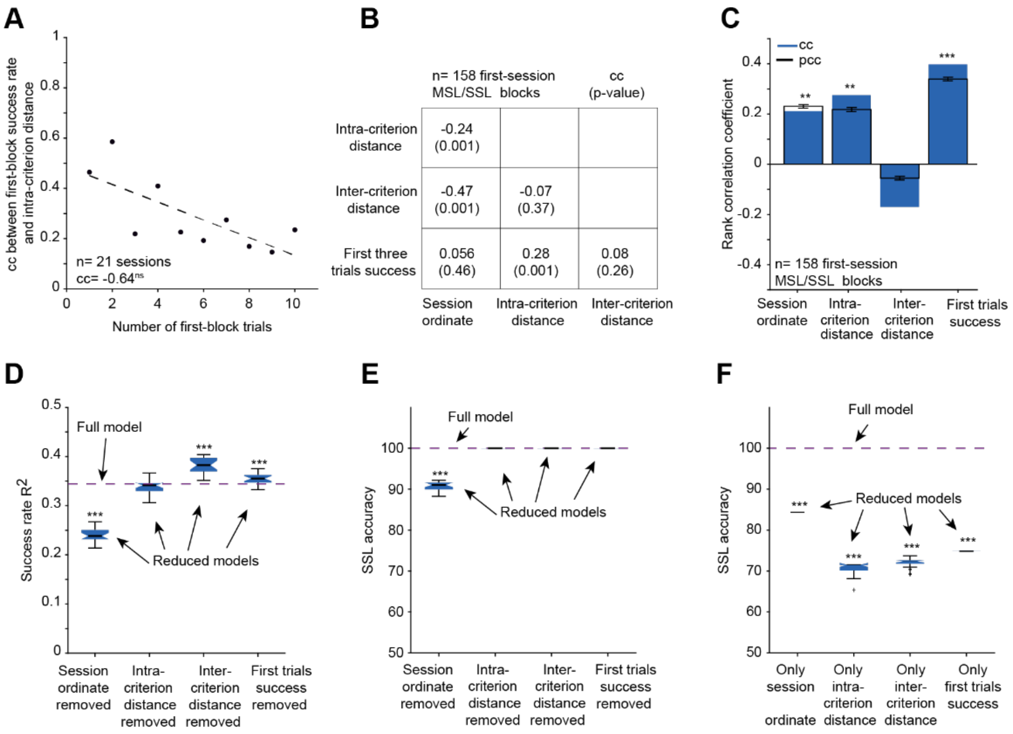

Figure S2. Success in the first three testing trials is correlated with DC difficulty. (**A**) Success rates during the first 1, 2, …, or 10 trials of SSL and MSL sessions is correlated with intra-criterion distance. cc, rank correlation coefficient. Here and in **C**, ns/*/**/***: p>0.05/p<0.05/p<0.01/p<0.001, permutation test. (**B**) Correlation matrix for the three features used in **Fig. 4B-G** (session ordinate, intra-criterion distance, inter-criterion distance) and a fourth feature, the success rate during the first three trials. p-value, permutation test. (**C**) cc's and pcc's between success rate and the four features described in **B**. Error bars, SD. (**D**) Variance in block success rate (R^2) explained by cross-validated support vector regression models. ***: p<0.001, Bonferroni-corrected U-test between the R^2 of the full model and R^2 of every reduced model, obtained by removing one feature. (**E**) Accuracy in predicting SSL using cross-validated support vector classification using the four-feature (full) model; and reduced three-feature models. Here and in **F**, ***: p<0.001, Bonferroni-corrected U-test between the accuracy of the full and reduced models. (**F**) Accuracy in predicting SSL using single-feature models.
